# INTERRELATIONSHIPS AMONG COLLECTIVE PERSONALITY, ATTITUDES TOWARD INTERGENERATIONAL SUPPORT, AND CO-RESIDENCE

**DOI:** 10.1093/geroni/igz038.1709

**Published:** 2019-11-08

**Authors:** Yooumi Lee

**Affiliations:** Syracuse University, Syracuse, New York, United States

## Abstract

This study investigates if collective personality influences positive attitude towards family caregiving and support for elderly parents, which, in turn, engenders positive attitude toward intergenerational coresidence. To test the hypothesis, the author examines the interconnections among collective personality; attitude towards family caregiving, and attitude towards support for aged parents; and attitude towards intergenerational coresidence in the U.S. The survey data is collected from total 67 undergraduate students in classroom environment. Particular attention is given to their attitude towards family caregiving and aged parents support. Results show that those three predictors are positively correlated with the attitude towards intergenerational coresidence i.e. respondents with more positive attitude towards family caregiving and aged parents support and with collective personality are more likely to have positive attitude towards intergenerational coresidence. Among the three predictors, collective personality is positively connected to the attitudes towards family caregiving and support for aged parents. A positive correlation is also observed between two attitude predictors i.e. respondents with more positive attitudes towards the family caregiving are likely to have positive attitude towards the support for aged parents and vice versa. The results highlights the important influence of collective personality, and attitude towards family caregiving and support for older parents on the intergenerational coresidence, even in the individualistic society.

